# Effects of a 12-Month Hybrid (In-Person + Virtual) Education Program in the Glycemic Status of Arab Youth

**DOI:** 10.3390/nu14091759

**Published:** 2022-04-22

**Authors:** Nasser M. Al-Daghri, Osama E. Amer, AbdulAziz Hameidi, Hanan Alfawaz, Mohammed Alharbi, Malak N. K. Khattak, Abdullah M. Alnaami, Naji J. Aljohani, Ghadah Alkhaldi, Kaiser Wani, Shaun Sabico

**Affiliations:** 1 Chair for Biomarkers of Chronic Diseases, Biochemistry Department, College of Science, King Saud University, Riyadh 11451, Saudi Arabia; oamer1@ksu.edu.sa (O.E.A.); mkhattak@ksu.edu.sa (M.N.K.K.); aalnaami@ksu.edu.sa (A.M.A.); kwani@ksu.edu.sa (K.W.); ssabico@ksu.edu.sa (S.S.); 2 Saudi Diabetes Charity, Riyadh 12721, Saudi Arabia; alsukkary@gmail.com; 3 Department of Food Science and Nutrition, College of Food Science and Agriculture, King Saud University, Riyadh 11495, Saudi Arabia; halfawaz@ksu.edu.sa; 4 Diabetes Centres and Units Administration, Ministry of Health, Riyadh 11176, Saudi Arabia; myal-harbi@moh.gov.sa; 5 Obesity Endocrine and Metabolism Center, King Fahad Medical City, Riyadh 11525, Saudi Arabia; najij@hotmail.com; 6 Department of Community Health Sciences, College of Applied Medical Sciences, King Saud University, Riyadh 11451, Saudi Arabia; ghalkhaldi@ksu.edu.sa

**Keywords:** diabetes, adolescents, pediatrics, arab, obesity

## Abstract

This 12-month school-based intervention study investigated the effects of hybrid educational lifestyle modifications on glycemic control among Saudi youth with different glycemic statuses. A total of 2600 Arab adolescents aged 12–18 years were recruited from 60 randomly selected schools. Anthropometrics, blood glucose, and HbA1c were measured pre- and post-intervention. Participants were grouped according to baseline HbA1c into normal, prediabetes, and diabetes groups. All participants received lifestyle education at base line and at every 3-month interval to improve diet and exercise behavior. Diabetic and prediabetic participants received a tailored lifestyle intervention. Post-intervention, 643 participants were analyzed as follows: 20 participants from the diabetes group, 39 from prediabetes, and 584 from the normal group. A modest but significant improvement in the glycemic status of diabetic and prediabetic participants was observed, but not in the normal group. In the diabetes group, 11 (55%) participants achieved normal HbA1c levels, 5 had prediabetes levels, and only 4 remained within diabetes HbA1c levels. In the prediabetes group, 34 (87.2%) participants achieved normal HbA1c levels, while 2 (5.1%) participants remained prediabetic and 3 (7.7%) had diabetes HbA1c levels (*p* < 0.001). This hybrid lifestyle intervention program modestly reduces the risk of T2DM among youth with elevated HbA1c levels. The challenge of sustaining interest in adopting lifestyle changes for a longer duration should be addressed in further studies in this population.

## 1. Introduction

Widespread obesity and pronounced lifestyle changes have led to the emergence of diabetes mellitus as a global public health challenge in children and adolescents [1,2]. In the past, type 1 diabetes (T1D) was the predominant type among children, nonetheless, in the past 2 decades, type 2 diabetes mellitus (T2DM) has taken the lead [3,4]. However, in some populations, T1D remains the predominant type among pediatrics, even though T2DM is rising. The prevalence of diabetes has dramatically increased between 1990 and 2008, where the annual increase in the T1D incidence was nearly doubled from 2.8% to 4.0% per year worldwide, whereas the prevalence of T2DM has increased 10 times in children 6–12 years old and almost doubled in adolescents from 7.3 to 13.9/100,000 between 1967 and 1997 [4]. Additionally, studies have demonstrated higher mortality in T2DM than in T1D [4,5].

The challenge of diabetes in youth could be attributed to the delayed diagnosis, early and late complications, increasing prevalence, psychosocial issues, management in school settings, and other factors which could interfere with glycemic control. According to IDF, Saudi Arabia has one of the highest prevalences of T1D in children, and recent studies have reported an incidence of 27.5 per 100,000 with 17% annual increase; moreover, as a child gets older the prevalence is expected to increase to 159 per 100,000 and [6,7].

Obesity is a well-known risk factor for T2DM in children and adolescents, which is a result of physical inactivity and high calorie intake, in addition to a sedentary lifestyle [8]. In the United States, the National Health and Nutrition Examination Survey (2015–2016) has reported a prevalence of obesity at 18.5% among U.S. children and adolescents aged 2–19 years [9]. Since most T2DM children and adolescents were found to be obese at the diagnosis [10], there is a need for obesity prevention programs, along with early detection of T2DM. In 2010, the U.S. preventative services task force recommended that at age of six years, young children should be screened for obesity with the endorsement of physical activity, behavioral modification, and moderate to intense dietary restrictions among obese children [11].

Saudi Arabia has had a widespread increase in pediatric obesity; in 2010, a Saudi National Diabetes Registry-based study on 19,317 children and adolescents aged 5–18 years (50.8% boys), reported an overall prevalence of overweight, obesity, and severe obesity at 23.1%, 9.3%, and 2%, respectively [12]. This prevalences of overweight and obesity among Saudi children were increased in 2014 to be 7.3% and 17.4% in boys, respectively. In girls, they were reported to be 12.4% and 20.9%, respectively [13]. Moreover, a recent cross-sectional, school-based national study included 12,575 Saudi children and adolescents aged 12–18 years, who demonstrated a prevalence of obesity of 15.9% [14]. It has worsened again in 2016 among Saudi children aged 5–19 years in a report from the World Health Organization (WHO), in which the prevalences of obesity and overweight were 17.4% and 35.6%, respectively, [15]. A recent study in the Riyadh region on 7930 children (aged 6–16 years) indicated that the prevalences of overweight and obesity in boys were 12% and 18.4%, respectively, while in girls they were 14.2% and 18%, respectively [16]. Furthermore, a 2015 epidemiologic study conducted in Riyadh among 23,523 Saudi children and adolescents revealed that the overall prevalence of pediatric DM was 10.4% based on fasting blood glucose >125 mg/dL, while only 0.07% were known to have T2DM [17]. In addition, a recent study conducted in Riyadh city among children with diabetes found that 64% were overweight or obese [18].

Therefore, it is essential to establish an effective diabetes self-management strategy, which should consider lifestyle changes in addition to children’s behaviors and attitudes toward diabetes prevention and management. However, such studies of diabetes management among children and adolescents are limited. Therefore, this study aims to assess, analyze, and draw conclusions about the effectiveness of school-based educational intervention programs promoting healthy diet and physical activity in a cohort of Saudi school-attending children and adolescents aged 12–18 years on the glycemic status.

## 2. Materials and Methods

### 2.1. Participants

**Figure 1:**
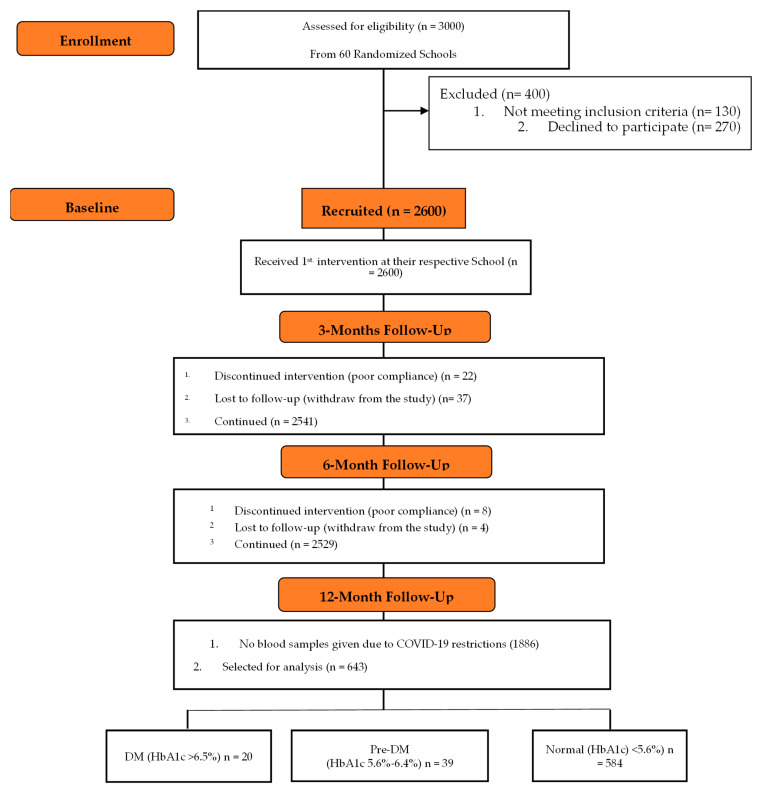
Flow chart of the study participants describing their participation and allocation.

At the start of the study, a total of 2600 school-attending Saudi children and adolescents aged 12–18 years (58% girls) were recruited from 60 randomly selected preparatory and high schools in Riyadh City, Saudi Arabia, in collaboration with the Chair for Biomarkers of Chronic Diseases, King Saud University, and the Saudi Charitable Association of Diabetes (SCAD), Riyadh, Saudi Arabia. This study included healthy children and adolescents, whether they were overweight, obese, or not. Those who were known cases of type 1 diabetes (T1DM) and/or who were on insulin therapy were excluded. Written informed consent was obtained from parents and assent from students. Post-intervention, only 643 participants were able to return for follow-up. All measurements were done twice (baseline and follow-up). A flow chart describing the study population is provided in Figure 1.

### 2.2. Intervention

All the intervention educational sessions took place at schools.

All participants were given diet and exercise education by certified dietitians, including standardized topics such as discontinuing juice/soda intake, bringing low-fat lunches to school, and decreasing portion sizes. Sedentary activities were discouraged, and physical activities that the child enjoyed were encouraged. To provide consistent education across sites, all the study staff were trained and were supplied with the exact educational materials. Participants were followed for 12 months with a 3-month interval during the study.

The first educational session took place in the school’s classrooms, and students were informed about T2DM risk factors, its pathogenesis, and the role of dietary restriction and increased physical activity in delaying the onset of T2DM. All were advised to modify their lifestyle through shifting to a healthy diet and implementing good exercise behaviors, to increase physical activity, and to reduce weight if they were overweight or obese. They also received information about recommended lifestyle changes in the form of pamphlets, booklets, infographic videos, and gamification. In the follow-up, participants were educated every 3 months through educational sessions about the lifestyle modifications necessary to prevent T2DM. These educational sessions were done by certified endocrinologists and nutritionists, and it was planned to take place at the schools. Due to COVID-19 lockdown, the follow-up educational activities (every 3 months) were done through a virtual meeting platform (Zoom), and the social media platforms WhatsApp, Telegram, Facebook, and Twitter.

Diabetes and prediabetes participants had lifestyle modifications tailored to each participant according to their lifestyle through the phone, using a diary from a registered dietitian. The suggested lifestyle changes were implemented previously at the different diabetes prevention programs conducted in KSA and are as follows: reducing body weight by at least by 5%, decrease fat intake (30% of total energy and 10% saturated fat), increase fiber intake to 15 g/1000 kcal, and lastly, exercising over 150 min/week or 30 min/day at moderate intensity [19,20,21]. Participants were educated about the effect of exercise on the regulation of blood glucose and were prescribed aerobic exercise of 30 min five times per week (e.g., bicycling, swimming, badminton, walking, etc.), whichever could be applicable during the COVID-19 lockdown. Based on their health conditions or lifestyle, the frequency, duration, and exercise type were personalized.

### 2.3. Anthropometric Measurements

The participants were instructed to come to their respective schools in a 10-h overnight fasting state. Anthropometric measurements were collected by trained nurses, including weight (cm), height (cm), body mass index BMI (kg/m^2^), hip (cm), and waist (cm) circumferences, while systolic and diastolic blood pressures were measured as the average of two readings with a 15-min interval, using pediatric cutoffs appropriate for children’s sizes as carried out in previous studies [22,23]. Overweight and obese children were classified according to sex, age and BMI percentile for children as done previously [24].

### 2.4. Biochemical Analyses

Fasting blood samples were collected by trained nurses at baseline and after intervention. Baseline HbA1c levels were used to stratify participants into three groups: normal, prediabetes, and diabetes groups based on the American Diabetes Association criteria (diabetes, HbA1c ≥6.5% (≥48 mmol/mol); prediabetes, A1C 5.7–6.4% (39–47 mmol/mol) <5.7% (<39 mmol/mol) [25]. Baseline and post-intervention HbA1c were analyzed twice for every blood sample using one of the National Glycohemoglobin Standardization Program (NGSP) certified methods, the D-10 Hemoglobin Testing System, (ion-exchange high-performance liquid chromatography) (Bio-Rad Laboratories, Hercules, CA, USA).

### 2.5. Definition of DM

We used the American Diabetes Association HbA1c cutoff criteria for the definition of DM status [HbA1c ≥6.5% (≥48 mmol/mol), pre-DM (HbA1C 5.7–6.4% (39–47 mmol/mol)] and normoglycemic (HbA1c <5.7% (<39 mmol/mol)) [25]. No additional tests were done for the classification of DM.

### 2.6. Physical Activity

Physical activity was measured using the International Physical Activity Questionnaire—Short Form (IPAQ-SF), which contains seven questions [26]. The official Arabic short version of the International Physical Activity Questionnaire has been validated and used in Saudi Arabia adult population studies [27,28,29,30].

### 2.7. Data Analysis

Data were analyzed using SPSS (version 22.0 Chicago, IL, USA). Continuous data were presented as mean ± standard deviation (SD) for normal variables and non-Gaussian variables were presented in median (1st and 3rd) percentiles. Categorical data were presented as frequencies and percentages (%). All continuous variables were checked for normality using the Kolmogorov–Smirnov test. Non-Gaussian variables were log-transformed prior to parametric analysis. Paired *t*-test and Wilcoxon sign tests were used to compare mean and median differences and one-way analysis of variance (ANOVA) was performed at baseline. The McNemar test was performed for physical activity, obesity, and T2DM status pre- and post-intervention. Multivariate repeated measure ANCOVA was performed between and within groups, adjusted for age and BMI. Significance was set at *p* < 0.05.

## 3. Results

A total of 643 (300 boys) out of 2600 participants (24.7% response rate) were able to provide their follow-up fasting blood samples at the Saudi Charitable Association of Diabetes (SCAD), Riyadh, Saudi Arabia. Table 1 displays our study population’s characteristics at baseline, stratified by sex. Height, weight, BMI, BMI Z-score, fasting blood glucose, and HbA1c were significantly higher in boys than girls. Girls were significantly more active than boys (53.6% vs. 39.0%, *p* < 0.001). Obesity was more prevalent in boys than in girls (25.3% vs. 10.8%, *p* < 0.001).

Pre- and post-intervention clinical characteristics of the three study groups are displayed in Table 2. Group comparisons demonstrated that after 12 months, as expected, significant increases in height were observed in all groups, as they were in a growing phase. Significant decrease in BMI over time was only observed in normal and prediabetes groups (*p*-values <0.001). This increase, however, was not observed in the BMI Z-scores of all groups. Fasting glucose and HbA1c significantly increased over time only in the normal group (*p* < 0.001). No significant change in fasting glucose over time was observed in either the prediabetes or T2DM groups. For the main outcome, there was a significant decrease in HbA1c values post-intervention in both prediabetes (HbA1c: 5.3 ± 0.9 post-intervention vs. 5.9 ± 0.2 at baseline, *p* < 0.001) and T2DM groups (HbA1c: 5.9 ± 1.3 post-intervention vs. 7.4 ± 0.9 at baseline, *p* < 0.001). Group analysis indicated that HbA1c significantly decreased in favor of the T2DM group [mean HbA1c change −1.6% (95% confidence interval, CI, −2.1–−1.0); *p* < 0.001] even after adjusting for sex, age, and BMI Z-score.

At baseline, we divided our cohort of participants based on their BMI into three groups: 106 (17.4%) obese, 111 (18.3%) overweight, and 391 (64.3%) normal BMI values. Table 3 illustrates the change in obesity status in all three groups of our study. After intervention, results indicated an overall improvement in the obesity status. 38 (35.8%) out of 106 obese participants shifted to be overweight after intervention, and 3 (2.8%) turned into normal status, while 65 (61.3%) stayed obese. In the overweight group, 54 (48.6%) participants out of 111 at baseline turned to normal BMI values after intervention and 55 (49.5%) stayed in the overweight status, while only 2 (1.8%) participants became obese after intervention (*p* < 0.001). Lastly, in the normal BMI group, 372 (95.1%) out of 391 participants stayed normal as baseline, and 19 (4.9%) participants turned to overweight status after intervention (*p* < 0.001).

Changes in the prevalence of DM and physical activity are displayed in Table S1. In the diabetes group, 11 (55.0%) diabetes participants out of 20 at baseline reversed to normal HbA1c levels after intervention and 5 (25.0%) improved to prediabetes HbA1c levels, while only 4 (20.0%) participants remained in the diabetes status. In the prediabetes group, 34 (87.2%) out of 39 participants reversed to normal HbA1c levels after intervention, 2 (5.1%) participants shifted to prediabetes status, and 3 (7.7%) to diabetes status. In the normal group, 475 (81.3%) out of 584 participants stayed normal after intervention, while 60 (10.3%) participants shifted to the prediabetes status and 49 (8.4%) became diabetic according to their HbA1c levels. Lastly, the cross-tabulation of participants’ diabetes against obesity status over time is displayed in Table S2. Data indicated no improvement in BMI status was prevalent among participants with T2DM post-intervention than baseline. There was a significant improvement in BMI status among prediabetes participants over time (*p* = 0.03). In contrast, among those with normal glycemic status, 5% became overweight post-intervention (*p* < 0.001).

## 4. Discussion

This 12-month school-based intervention program significantly decreased the prevalence of diabetes and prediabetes among our study cohort of school children and adolescents. Our study demonstrated that lifestyle modification significantly improved glycemic control (HbA1c) in children and adolescents at risk of T2DM. This is in agreement with previous studies in both children [31,32] and adults [33,34]. Herein, intervention with diet and exercise education has reversed the prediabetes and diabetes statuses to normoglycemia in our cohort. This outcome is similar to the results of the U.S. randomized controlled trial on 3234 overweight adults [35], which promoted lifestyle modification as a recommended approach for subjects at risk of developing T2DM in all ages. A recent 6-month intervention study on obese children and adolescents with impaired glucose tolerance combined the treatment with metformin (which is an oral medication approved by FDA for the treatment of adolescents with T2DM) and lifestyle intervention, they found no difference between lifestyle intervention with or without metformin in normalizing glucose levels [36]. More randomized controlled trials support these findings [35,37].

In our study, we focused on the prediabetic and diabetic participants of our cohort, where we provided a personalized educational program to these participants, particularly the overweight and obese participants. This intervention resulted in a significant decrease in BMI among prediabetic and diabetic participants in our cohort, which is similar to previous studies for weight management in pediatrics [38,39]. In our study, we analyzed the change in BMI after intervention instead of the weight change, as our participants were still growing in height. The favorable changes in the indices of obesity in our study cohort are in line with results of the HEALTHY study, a large American school-based multicomponent intervention program for children at risk for obesity and T2DM [40]. Moreover, changes in body weight and composition after lifestyle interventions were also demonstrated previously [41,42] which contributed to improvements in insulin sensitivity.

The present study has some limitations. We did not actively monitor the participants’ physical activity and dietary intake. Therefore, we could not assess whether the modest weight loss among our study cohort was due to restrictions in diet, exercise, or both. Second was the high dropout rate in our study after intervention, thus limiting the actual effect of the intervention in the study duration. The high dropout rate could be due to the COVID-19 lockdown and the restrictive regulations during study period. However, this large dropout might also suggest that sustaining interest in lifestyle change is difficult to do, particularly among our cohort of youth, and they may revert to their previous habits in time.

Despite these limitations, these study findings are the first to observe whether a hybrid setting of in-person + virtual can elicit favorable effects in the glycemic status of Arab adolescents. Our results add to the current literature and have clinical value, given the ethnic and cultural variations in compliance to such a diabetes prevention intervention program. Improving and even normalizing the glycemic status among diabetic and prediabetic children and adolescents, even with a hybrid approach, is an effective strategy to prevent the development of T2DM in people at risk, or even reverse T2DM status to normal. Moreover, it was demonstrated previously that the positive effects of lifestyle modification approaches can be long-lasting [43].

## 5. Conclusions

Results of this 12-month lifestyle intervention study demonstrate that improving glycemic status is achievable through lifestyle modifications delivered through different platforms among Arab adolescents. The reversion to normal status was in agreement with the improvement in participants’ BMI. Further studies are needed to determine whether lifestyle interventions can be translated into sustainable improvements in clinical practice that reduce the risk of developing T2DM.

## Figures and Tables

**Table 1:** General characteristics of all participants at baseline.

Parameters	All	Boys	Girls	*p*-Value
N	643	300	343	
Age	14.8 ± 1.7	14.7 ± 1.6	14.9 ± 1.8	0.13
Height (cm)	156.2 ± 9.3	158.2 ± 10.5	154.4 ± 7.6	<0.001
Height Z-score	0.0 ± 1.0	0.22 ± 1.1	−0.19 ± 0.8	<0.001
Weight (kg)	56.4 ± 17.5	60.1 ± 20.3	53.4 ± 14.1	<0.001
Weight Z-score	0.0 ± 1.0	0.21 ± 1.2	−0.18 ± 0.8	<0.001
BMI (kg/m^2^)	23.0 ± 6.0	23.7 ± 6.7	22.4 ± 5.4	0.009
BMI Z-score	0.0 ± 1.0	0.12 ± 1.1	−0.10 ± 0.9	0.009
Glucose	5.21 ± 0.8	5.30 ± 1.1	5.13 ± 0.5	0.008
HbA1c (%)	5.11 ± 0.6	5.21 ± 0.6	5.02 ± 0.6	<0.001
Physical Activities Status N (%)				<0.001
Yes	301 (46.8)	117 (39.0)	184 (53.6)	
No	342 (53.2)	183 (61.0)	159 (46.4)	
DM N(%)				0.45
Normal	584 (90.8)	269 (89.7)	315 (91.8)	
Pre-DM	39 (6.1)	22 (7.3)	17 (5.0)	
Diabetes	20 (3.1)	9 (3.0)	11 (3.2)	
Obesity Status N (%)				<0.001
Normal	391 (64.2)	159 (57.4)	232 (69.9)	
Overweight	112 (18.4)	48 (17.3)	64 (19.3)	
Obese	106 (17.4)	70 (25.3)	36 (10.8)	

Note: Data presented N (%) and mean ± SD, *p*-value significant at 0.05 and 0.01 level.

**Table 2:** General Characteristic of the subjects at baseline and after intervention.

Parameters	Normal			Prediabetes			Diabetes			Between Group Adjusted *p*-Value
	Baseline	Follow-Up	*p*-Value	Baseline	Follow-Up	*p*-Value	Baseline	Follow-Up	*p*-Value	
Age (Years)	14.8 ± 1.7			15.3 ± 1.7			15.3 ± 1.3			
Weight (kg)	55.8 ± 17.1	57.8 ± 15.6	<0.001	65.8 ± 21.5	68.1 ± 19.1	<0.001	54.9 ± 14.3	57.3 ± 13.3	<0.001	
Weight Z-score	−0.03 ± 0.9	−0.04 ± 0.9	0.39	0.54 ± 1.2	0.61 ± 1.2	0.01	−0.09 ± 0.8	−0.07 ± 0.8	0.43	
Height (cm)	156.0 ± 9.3	164.6 ± 6.5	<0.001	156.4 ± 9.9	166.8 ± 7.3	<0.001	160.1 ± 7.3	166.4 ± 6.9	0.003	
Height Z-score	−0.02 ± 1.0	−0.03 ± 0.9	0.78	0.02 ± 1.1	0.32 ± 1.1	0.13	0.42 ± 0.8	0.25 ± 1.0	0.50	
BMI (kg/m^2^)	22.8 ± 5.9	21.4 ± 5.7	<0.001	26.4 ± 6.7	24.3 ± 6.5	<0.001	21.4 ± 4.9	20.6 ± 4.6	0.14	
BMI Z-score	−0.02 ± 0.04	−0.03 ± 0.04	0.81	0.48 ± 0.19	0.55 ± 0.18	0.29	−0.17 ± 0.2	−0.27 ± 0.2	0.28	
Glucose	5.2 ± 0.5	5.7 ± 2.8	<0.001	5.4 ± 0.9	5.5 ± 2.2	0.89	6.6 ± 3.4	6.6 ± 3.4	0.99	0.02
HbA1c (%)	5.0 ± 0.3	5.3 ± 1.3	<0.001	5.9 ± 0.2	5.3 ± 0.9	<0.001	7.4 ± 0.9	5.9 ± 1.3	<0.001	0.01

Note: Data presented as mean ± SD for baseline and follow-up. *p*-value is obtained from repeated measures ANCOVA adjusted for (age, sex, BMI Z-score). *p* < 0.05 is considered significant.

**Table 3:** Percentage (%) change in obesity and physical activity status.

BMI Status	Baseline		Post-Intervention			*p*-Value
			Normal	Overweight	Obese	
Normal	391 (64.3)		372 (95.1)	19 (4.9)	0 (0.0)	<0.001
Overweight	110 (18.3)		53 (48.6)	55 (49.5)	2 (1.8)	
Obese	105 (17.4)		3 (2.8)	38 (35.8)	65 (61.3)	
Physical Activity (N)						
Normal	High PA	34	32 (94.1)	2 (5.9)	0	0.13
	Moderated PA	143	140 (97.9)	3 (2.1)	0	
	Low PA	205	191 (93.2)	14 (6.8)	0	
Overweight	High PA	13	8 (61.8)	5 (38.5)	0	0.10
	Moderated PA	31	20 (64.5)	11 (35.5)	0	
	Low PA	66	25 (37.9)	39 (59.1)	2 (3.0)	
Obese	High PA	10	0	6 (60.0)	4 (40.0)	0.25
	Moderated PA	35	2 (5.7)	14 (40.0)	19 (54.3)	
	Low PA	60	1 (1.7)	18 (30.0)	41 (68.3)	

Note: Data presented N (%). *p*-value significant at 0.05 and 0.01 level using McNemar test.

## Data Availability

The data presented in this study are available on request from the corresponding author. The data are not publicly available due to privacy protection.

## Supplementary Materials

The following supporting information can be downloaded at: https://www.mdpi.com/article/10.3390/nu14091759/s1, Table S1: Baseline characteristics of all recruited participants. Table S2: Cross tabulation BMI and diabetes status at baseline and post-intervention.
