# Supplementary material for: Effects of a 12-Month Hybrid (In-Person + Virtual) Education Program in the Glycemic Status of Arab Youth
Source: Nutrients. 2022 Apr 22;14(9):1759. doi: 10.3390/nu14091759 (PMC9103307; doi:10.3390/nu14091759)
Supplement: Supplementary file 1 [file nutrients-14-01759-s001.zip › nutrients-1656818-supplementary.pdf]

Supplementary Table S1: Percentage (%) change in Diabetes and Physical Activity (PA) status.

Parameters	Baseline DM Status		Post-Intervention			P-Value
			Normal	Pre-DM	DM	
DM	20 (3.1)		11 (55.0)	5 (25.0)	4 (20.0)	<0.001
Pre-DM	39 (6.1)		34 (87.2)	2 (5.1)	3 (7.7)	
Normal	584 (90.8)		475 (81.3)	60 (10.3)	49 (8.4)	
**Physical Activity (N)**						
**DM**	High PA	5	4 (80.0)	1 (20.0)	0	0.55
	Moderated PA	9	4 (44.4)	3 (33.3)	2 (22.2)	
	Low PA	6	2 (33.3)	2 (33.3)	2 (33.3)	
**Pre-DM**	High PA	6	6 (100)	0	0	0.20
	Moderated PA	14	14 (100)	0	0	
	Low PA	19	14 (73.7)	2 (10.5)	3 (15.8)	
**Normal**	High PA	46	39 (84.8)	5 (10.9)	2 (4.3)	0.57
	Moderated PA	200	165 (82.5)	22 (11.0)	13 (6.5)	
	Low PA	327	264 (80.7)	31 (9.5)	32 (9.8)	

Note: Data presented as N (%). PA, physical activity; P-value significant at 0.05 using McNemar test.

Supplementary Table S2: Cross-tabulation BMI and diabetes status at baseline and post-intervention.

Diabetes Status	Baseline BMI	N (%)	Post-intervention			P-Value
			Normal	Overweight	Obese	
**DM**	Normal	14 (70)	13 (92.9)	1 (7.1)	0	0.25
	Overweight	4 (20)	4 (100.0)	0	0	
	Obese	2 (10)	0	1 (50.0)	1 (50.0)	
**Pre-DM**	Normal	15 (40.5)	15 (100)	0	0	0.03
	Overweight	9 (23.1)	6 (66.7)	3 (33.3)	0	
	Obese	13 (33.3)	1 (7.7)	2 (15.4)	10 (76.9)	
**Normal**	Normal	362 (62)	344 (95)	18 (5.0)	0	<0.001
	Overweight	99 (17)	45 (45.5)	52 (52.5)	2 (2)	
	Obese	91 (15.6)	2 (2.2)	35 (38.5)	54 (59.3)	

Note: Data presented N (%). P-value significant at 0.05 using Fisher exact test and McNemar test.
